# Lymphoid follicular hyperplasia arising from the chest wall presenting as a substantial mass

**DOI:** 10.1002/rcr2.1417

**Published:** 2024-07-14

**Authors:** Naoya Kitamura, Keitaro Tanabe, Toshihiro Ojima, Koichiro Shimoyama, Akira Noguchi, Kenichi Hirabayashi, Tomoshi Tsuchiya

**Affiliations:** ^1^ Department of Thoracic Surgery Toyama University Hospital Toyama Japan; ^2^ Department of Diagnostic Pathology Toyama University Hospital Toyama Japan

**Keywords:** benign tumour, chest wall, lymphoid follicular hyperplasia, lymphoproliferative disease, thoracoscopic surgery

## Abstract

Lymphoid follicular hyperplasia (LFH) is a benign lymphoproliferative disease. Although it can occur within the thoracic cavity, LFH originating from the chest wall has not been reported. A 79‐year‐old woman was incidentally found to have a well‐defined mass on the left posterior chest wall during a preoperative examination for aortic valve replacement. The mass had slowly grown over 6 years. Thoracoscopic surgical resection was performed without complications. Pathological examination ruled out lymphoproliferative diseases, such as Castleman disease or malignant lymphoma, and a diagnosis of LFH was made. Although LFH generally has a good prognosis, surgical resection is recommended for diagnostic and therapeutic purposes owing to the possibility of malignancy masquerading as a reactive lesion. This is the first report of an LFH arising from the chest wall with imaging findings similar to other benign tumours. Its potential as a differential diagnosis for tumours with similar imaging findings is highlighted.

## Graphical abstract

Lymphoid follicular hyperplasia, a rare benign lymphoproliferative disease, was found on the chest wall of a 79‐year‐old woman. Surgical resection confirmed the diagnosis, emphasizing lymphoid follicular hyperplasia as a potential alternative diagnosis for similar benign tumours.

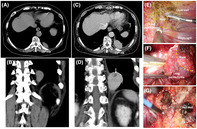

## INTRODUCTION

Lymphoid follicular hyperplasia (LFH) is a benign disease wherein B‐cells in the lymph nodes reactively proliferate and is the most common pattern of reactive lymphadenopathy. In the thoracic cavity, reactive lymph node enlargement originating from the lung parenchyma or anterior mediastinum has been reported.^1^, ^2^ Herein, we report the first rare case of LFH arising from the chest wall.

## CASE REPORT

**FIGURE 1:**
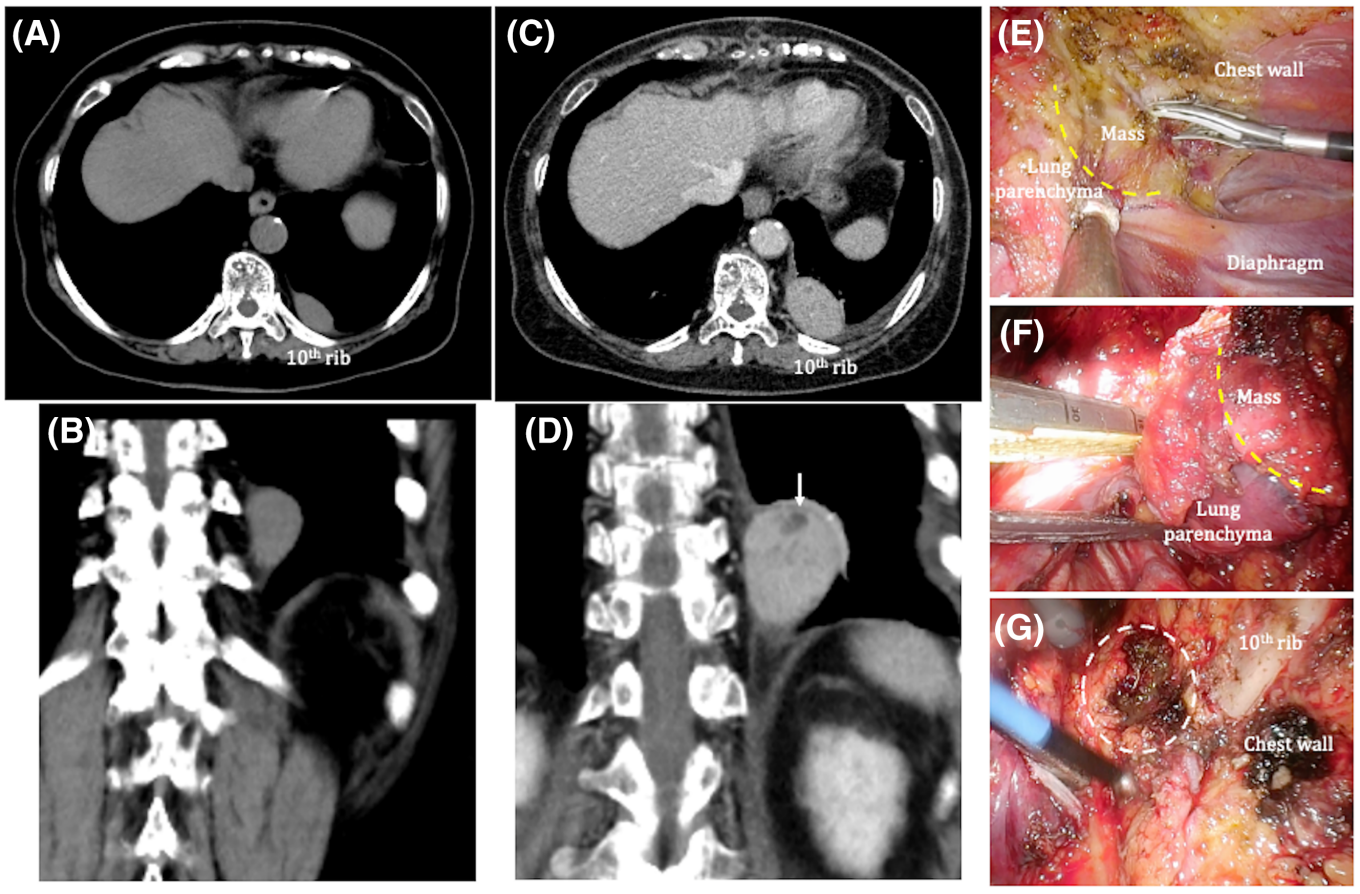
(A, B) Chest computed tomography images taken 6 years prior show a mass measuring 32 × 17 × 32 mm on the left posterior chest wall (near the joint of the head of the 10th rib) with well‐defined margins. (C, D) Contrast‐enhanced chest computed tomography images show a well‐defined mass measuring 41 × 25 × 41 mm on the left posterior chest wall (near the joint of the head of the 10th rib), with some areas of poor contrast enhancement (white arrow). (E, F) The mass is particularly adherent to the adjacent left lower lobe lung parenchyma (yellow dotted lines), necessitating concomitant resection. (G) Intraoperative image after resection shows the area where the mass was located (white dotted circle).

**FIGURE 2:**
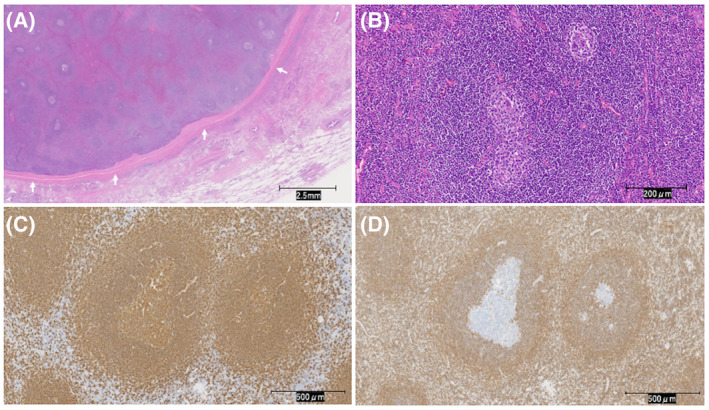
(A) The tumour is covered by a continuous capsule and contains variably sized and shaped follicles. There is no continuity with the lung parenchyma that was concomitantly resected (white arrows) (haematoxylin‐eosin staining, ×1). (B) The mass is composed of reactive lymphoid follicles. Germinal centres are surrounded by orderly marginal and mantle zones. Hyaline‐vascular or plasma cell types typical of Castleman disease are not seen (haematoxylin‐eosin staining, ×20). (C) CD20 (a B‐cell marker) is positive mainly in the follicular area (CD20, ×10). (D) bcl‐2 is negative in the follicular centre (bcl‐2, ×10).

A 79‐year‐old woman undergoing preoperative evaluations prior to aortic valve replacement for aortic stenosis was incidentally noted to have a 40‐mm‐sized mass on the left posterior chest wall on chest computed tomography (CT). Upon clinical chart review, a solid mass measuring 32 × 17 × 32 mm was seen on CT images 6 years prior (Figure 1A,B); it had been slowly growing and no treatment had been specifically administered. Blood tests showed no inflammatory reaction, and lactate dehydrogenase (LDH) levels were not elevated (white blood cells 8000/μL, neutrophils 58.6%, C‐reactive protein 0.06 mg/dL, LDH 221 U/L). Based on the morphology and growth rate of the mass, a benign tumour originating from the chest wall was suspected. Therefore, resection was not considered urgent, as we thought that simultaneous resection with cardiac surgery would cause excessive burden on the patient. Thus, we decided to resect the mass after the patient had achieved stability in the postoperative course of cardiac surgery. Contrast‐enhanced chest CT taken again after cardiac surgery identified a well‐defined mass measuring 41 × 25 × 41 mm (Figure 1C,D). Thoracoscopic resection of the mass was performed 6 months after aortic valve replacement. The thoracic cavity was extensively adherent; the mass was particularly firmly adherent to the lung; thus, part of the left lower lobe of the lung was concomitantly resected (Figure 1E–G). The mass was elastic and soft. The patient's postoperative course was uneventful; however, she was discharged on postoperative day 6 at her request to remain in the hospital for few more days to enable closer follow‐up. Pathologic examination revealed the mass to be lymph node tissue covered with fibrous membrane (Figure 2A). Increased numbers of reactive follicles of various sizes and shapes were noted. Hyaline‐vascular or plasma cell types typical of Castleman disease were not observed (Figure 2B). The germinal centre was positive for B cell marker CD20 (Figure 2C) and negative for bcl‐2, ruling out follicular lymphoma (Figure 2D). Additional history and physical examination revealed no underlying disease, such as rheumatoid arthritis, Sjögren's syndrome, or systemic lupus erythematosus. Based on these results, the patient was diagnosed with LFH and is currently under outpatient observation. Chest CT images taken 6 months after the surgery showed that the mass was completely excised and there were no findings suggesting recurrence.

## DISCUSSION

This rare case of LFH arising from the chest wall showed a mass similar to a benign posterior mediastinal tumour with very slow growth.

Several patterns of lymphoproliferative disease can occur in the thoracic cavity. Pulmonary nodular lymphoid hyperplasia typically arises from the lung parenchyma.^1^ It is commonly seen as a solitary pulmonary nodule and may present as a ground glass nodule^1^ or pleural indentations, or with mediastinal or hilar lymph node involvement.^3^ This hyperplasia can also occur in the anterior mediastinum, and LFH associated with thymic cysts has been reported.^2^ Castleman disease may also present with asymptomatic lymphadenopathy that shows a well‐defined mass on chest CT.^4^ Thus, a variety of lymphoproliferative diseases may occur within the thoracic cavity. Pathologically, LFH can be differentiated from lymphoproliferative diseases such as Castleman disease by the presence of small blood vessels in the lymph follicles,^4^ although differentiating these diseases is difficult based on CT images. Furthermore, it was suggested that there are no specific serum markers for lymphoid hyperplasia.^3^

There is no evidence suggesting that lymphoid hyperplasia regresses spontaneously.^3^ Therefore, surgical resection is considered the first‐line treatment for LFH to rule out potential malignancy.^1^, ^4^ Although the prognosis is generally favourable^1^, ^5^ and the patient had a slowly growing mass with no obvious malignant pathology, careful follow‐up is warranted because LFH may be a type of malignant lymphoma that mimics reactivity.^5^

To the best of our knowledge, this is the first report of LFH arising from the chest wall with imaging findings similar to other benign tumours. We emphasize its potential as a differential diagnosis for tumours with similar CT findings.

## AUTHOR CONTRIBUTIONS

Naoya Kitamura drafted and edited the manuscript. Keitaro Tanabe and Toshihiro Ojima performed a critical review of this report. Akira Noguchi and Kenichi Hirabayashi performed the pathological diagnosis and critical review of this report and provided advice regarding the pathological findings. Koichiro Shimoyama and Tomoshi Tsuchiya supervised the preparation of the case report. All authors have read and approved the final manuscript.

## CONFLICT OF INTEREST STATEMENT

None declared.

## ETHICS STATEMENT

The authors declare that appropriate written informed consent was obtained for the publication of this manuscript and the accompanying images.

## Data Availability

Data sharing is not applicable to this article as no new data were created or analyzed in this study.
